# Genetic Variability of *Plasmodium malariae dihydropteroate synthase* (*dhps*) in Four Asian Countries

**DOI:** 10.1371/journal.pone.0093942

**Published:** 2014-04-03

**Authors:** Naowarat Tanomsing, Mayfong Mayxay, Paul N. Newton, Francois Nosten, Christiane Dolecek, Tran Tinh Hien, Nicholas J. White, Nicholas P. J. Day, Arjen M. Dondorp, Mallika Imwong

**Affiliations:** 1 Department of Molecular Tropical Medicine and Genetics, Faculty of Tropical Medicine, Mahidol University, Bangkok, Thailand; 2 Lao-Oxford-Mahosot Hospital-Wellcome Trust Research Unit (LOMWRU), Microbiology Laboratory, Mahosot Hospital, Vientiane, Lao PDR; 3 Faculty of Postgraduate Studies, University of Health Sciences, Vientiane, Lao PDR; 4 Centre for Tropical Medicine, Nuffield Department of Medicine, Churchill Hospital, University of Oxford, Oxford, United Kingdom; 5 Shoklo Malaria Research Unit, Mahidol-Oxford Tropical Medicine Research Unit, Bangkok, Thailand; 6 Oxford University Clinical Research Unit, Hospital for Tropical Diseases, Ho Chi Minh City, Vietnam; 7 Mahidol-Oxford Tropical Medicine Research Unit, Faculty of Tropical Medicine, Mahidol University, Bangkok, Thailand; National University of Singapore, Singapore

## Abstract

The *dihydropteroate synthase* (*dhps*) genes of 44 *P. malariae* strains from four Asian countries were isolated. Only a limited number of polymorphisms were observed. Comparison with homologous mutations in other *Plasmodium* species showed that these polymorphisms are unlikely to be associated with sulfadoxine resistance.

## Introduction

Molecular characterizations of antifolate resistance in *Plasmodium falciparum* and *P. vivax* have revealed stepwise increase in antifolate resistance with additional mutations within the *dihydrotheorate synthase* (*dhps*) and *dihydrofolate reductase* (*dhfr*) genes [1], [2], [3], [4]. Although patients infected with *P. malariae* are usually not treated with antifolate drugs, exposure is still likely in endemic areas where sulfadoxine - pyrimethamine is used for treatment of falciparum malaria, because of the high frequency of mixed infections. The previously reported S114N mutation within the *dhfr* gene of *P. malariae* supports this [5], [6]. The *P. malariae* gene for *dhps*, the target for sulfadoxine, has not been studied to date.

## Materials and Methods

In this study, the *dhps* gene of 44 *P. malariae* strains from Asian clinical samples (34 from Thailand, 6 from Laos, 3 from Viet Nam, and 1 from Bangladesh) were isolated and analysed. Genomic DNA of *P. brasilianum,* a New World monkey malaria parasite considered to be genetically indistinguishable from *P. malariae*, purified from a cloned line maintained in Saimiri monkeys was obtained from MR4 (MR4-349). Samples were obtained from patients enrolled in a variety of previous treatment studies. All patients provided written informed consent. The protocol for this study was reviewed and approved from the Faculty of Tropical Medicine, Mahidol University, Thailand (reference no. MUTM2011-049-03). To design primers for isolation of the *dhps* gene from *P. malariae* and *P. brasilianum*, the nucleotide sequences of *dhps* gene from other human *Plasmodium* spp. were aligned, including *P. falciparum* (accession number XM_001349382), *P. vivax* (accession number XM_001617159), and *P. knowlesi* (accession number XM_002262146). A nested and seminested PCR approach was used to increase sensitivity of the amplification product. The sequences of primers, Mg^2+^concentrations, annealing temperatures, numbers of cycles, and sizes of products were individually determined for the different primer pairs shown in Table 1. Amplified PCR products were then cloned into the pCR 2.1 vector (Invitrogen, USA), and the plasmids purified from the bacterial clones were submitted for DNA sequencing.

**Table 1:** Primer sequences and PCR condition for isolation of *pppk-dhps* gene from *P. malariae* and *P. brasilianum*.

Primer name	Sequences (5′ to 3′)	Annealing temperature(°C)	MgCl_2_(mM)	No. of PCRcycle		Product size(bp)
				Nest1	Nest2	
DHPS_F70	GGAAC(G,A)AATGA(T,C)A(G,A)AA(G,A)(G,A)AAC	50	3	30		ca 800
DHPS_R800	CTGT(G,A)T(G,T)T(C,T)GT(G,A)TACACATGAGG					
DHPS_F120	GG(A,C)AAAAT(T,C)AT(T,C)AA(T,C)A(G,C)(T,G)TC(G,C)TAC	50	3		35	ca 700
DHPS_R800	CTGT(G,A)T(G,T)T(C,T)GT(G,A)TACACATGAGG					
DHPS_F500	GTTA(G,A)(G,A)AC(T,C)TTTGT(T,A)(G,A)A(T,A)GA(T,C)CC	50	3	30		ca 1,500
DHPS_R22	CTAA(C,A)ACGTC(G,A)TGAACTCT(G,T)AT(A,T)AG					
DHPS_F500	GTTA(G,A)(G,A)AC(T,C)TTTGT(T,A)(G,A)A(T,A)GA(T,C)CC	50	3		35	ca 1,000
DHPS_R16	GGATTTCC(C,T)CT(C,T)TT(G,A)TGCATT					
DHPS_MF900	GACACATTGAAGCAATTGAAAGA	52	3	30		ca 1,400
DHPS_PSR1	GTTTCTAA(A/C)ACGTC(A/G)TGAACTCT					
DHPS_MF17	GACATTAGCGCATGCACAAA	52	3		35	ca 700
DHPS_R22	CTAA(C,A)ACGTC(G,A)TGAACTCT(G,T)AT(A,T)AG					
DHPS_MF900	GACACATTGAAGCAATTGAAAGA	52	3		35	ca 700
DHPS_R16	GGATTTCC(C,T)CT(C,T)TT(G,A)TGCATT					
DHPS_MF17	GACATTAGCGCATGCACAAA	52	3		35	ca 900
DHPS_R2	AGCTGTAGGAAGCAAT(G,T)GCTA(G,A)(C,T)C					

## Results and Discussion

The partial *pppk-dhps* gene was isolated from both *P. malariae* (1,953 bp) and *P. brasilianum* (1,914 bp). The partial 1,953 bp *Pmpppk-dhps* included one intron (167 bp) at the carboxyl terminus coding region, which position and size was established by reverse-transcriptase PCR amplification and sequencing from corresponding mRNA. DNA sequences of *pppk-dhps* are available from 3 other species infecting humans: *P. falciparum*, *P. vivax* and *P. knowlesi.* In all species the *pppk-dhps* gene has two introns, one of which is located near the N-terminus coding region of the *pppk* gene and one near the C-terminal. This study revealed an 167 bp intron within *dhps* gene of *P. malariae.* AT content of the *pppk-dhps* gene from *P. malariae*, *P. brasilianum* and *P. falciparum* was high (72.2–76.5%), whereas this is low in *P. vivax* and *P. knowlesi* (56.8% and 62.2% respectively). The *pppk-dhps* gene of *P. falciparum* appeared to be located on chromosome 8, whereas in *P. vivax* and *P. knowlesi* this is on chromosome 14.

**Figure 1:**
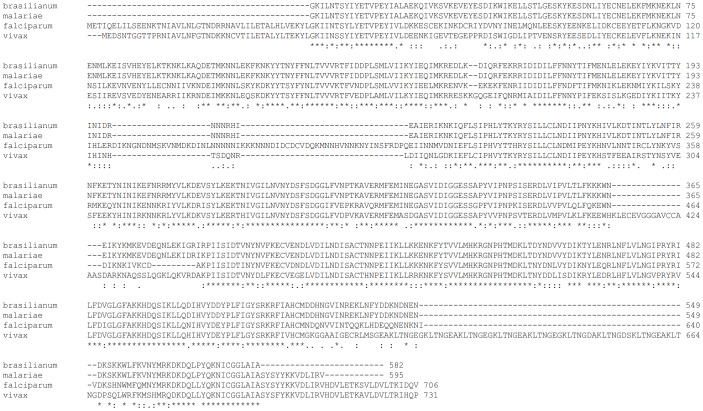
Deduced partial PPPK-DHPS amino acid sequences alignment.

Specific primers were designed to isolate the *Pmdhps* gene from all 44 *P. malariae* isolates. In the amplified product 1,140 bp (corresponding to 379 amino acids) of *Pmdhps* were analyzed, which revealed 2 and 3 positions of synonymous and nonsynonymous mutations which were predicted to change amino acids located outside the binding pocket of the enzyme for sulfadoxine. This was assessed through amino acid alignment with DHPS of *P. falciparum* and *P. vivax* (Figure 1), showing that equivalent variation in these other species are not associated with changes in the binding pocket for sulfadoxine, nor with sulfadoxine resistance. The 44 *Pmdhps* sequences could be categorized into 4 haplotypes; the wild type haplotype 1 was most prevalent (41/44 isolates; Table 2).

**Table 2:** Nonsynonymous mutations observed in *P. malariae dhps.*

DHPS amino acid residues*																
Organisms																No. of isolate
*P. falciparum*	S436A/F	A437G	K540E	A581G	A613S/T	P438	F580	R608	K609	Y663	N666	H688	T573	L516	E339	
*P. vivax*	S382	A383	K512	A553	V585	P384	F552	R580	K581	Y688	N691	H713	T509	L488	E285	
*P. knowlesi*	S382	A383	K514	A555	V587	P384	F554	R582	K583	Y711	N714	H737	T511	L490	E285	
*P. brasilianum*	S	A	K	A	A	P	F	R	K	Y	N	-	T	L	N	
*P. malariae haplotype 1*	S	A	K	A	A	P	F	R	K	Y	N	-	T	L	N	41#
*P. malariae haplotype 2*	S	A	K	A	A	P	F	R	K	Y	N	-	A	L	N	1 Thai
*P. malariae haplotype 3*	S	A	K	A	A	P	F	R	K	Y	N	-	T	F	N	1 Thai
*P. malariae haplotype 4*	S	A	K	A	A	P	F	R	K	Y	N	-	T	L	Y	1 Viet Nam

*Ten residues including 436,437,438,580,608,609,613,663,666,688 were predicted to contact with sulfadoxine in Pf with equivalent to Pv and other spp. while the first five residue were associated with sulfadoxine resistance. The last 3 residues were nonsynonymous mutation found in *P. malariae*.

# 32 Thai, 6 Lao PDR, 2 Viet Nam, 1 Bangladesh.

The current study investigated 27 isolates from which we have previously reported details on *dhfr* gene polymorphisms [6]. Two of these isolates contained the S114N-mutation within *dhfr*, which are predicted to confer antifolate resistant, and thus suggest antifolate drug pressure on the parasite populations. However, these *dhfr* mutations were not accompanied by mutations in the *dhps* gene thought to confer sulfadoxine resistance: one of these two samples contained wild type *dhps* gene, while the other strain showed 2 SNPs (one synonymous and one nonsynonymous). The nonsysnonymous mutation was located at the equivalent position 516 in *P. falciparum* and 488 in *P. vivax*, which does not code for the enzyme binding pocket. The *dhfr* gene sequences were then assessed in the 17 *P. malariae* isolates from Thailand, none of which showed mutations, and all were classified as haplotype 4 [6]. Thus far all available published and unpublished *Pmdhfr* sequences [5], [6] show a high prevalence of the wild type gene. Homology with other *Plasmodium* species suggest that the initial mutation associated with drug pressure may be at position S114N. In order to assess this point mutation more efficiently (analogous to S108N and S117N in *P. falciparum* and *P. vivax)*, a PCR-RFLP method was developed and investigated. Patterns of wild type S114 contain two bands of 401 bp and 172 bp+168 bp product, and mutant type 114N showed two bands of 569 bp and 172 bp product. Forty-four samples of *P. malariae* were tested and PCR-RFLP results were in accordance with the sequence data. Using PCR-RFLP, we analysed 17 isolates recently collected in Thailand which revealed no mutations, with all strains classified as the common haplotype 4 [6].

Comparing homology of *dhps* genes isolated from *P. falciparum*, *P. vivax, P. knowlesi* and *P. malariae* has some limitations. Amino acid alignment of DHPS in each *Plasmodium* spp. determined equivalent position of nonsynonnymous mutations in association to sulfadoxine resistance in *P. falciparum* and *P. vivax* (Table 2). All 44 isolates of *P. malariae* showed wild type amino acids at these equivalent positions, whereas the three nonsynonymous mutations present in three *P. malariae* isolates have not been described in these other species. They are equivalent to T537, L516 and E339 in *P. falciparum* (Table 2). Amino acids found in *P. vivax* and *P. knowlesi* at these three residues are the same as in *P. falciparum*, suggesting that these three residues are conserved among *Plasmodium* spp. It is therefore interesting to construct an in silico comparative structure model to further explore whether the nonsynonymous mutations found in *P. malariae* at these positions have an effect on molecular docking of sulfadoxine.

Our findings suggest that drug pressure of sulphadoxine-pyrimethamine on *P. malariae* in the region has been lower than on *P. falciparum* or *P. vivax*. Mutations in the PmDHPS and PmDHFR genes are rare in Thailand, suggesting absence of selection of SP resistant parasite populations. We plan to extend our observations to *P. malariae* isolates collected from African countries where SP drug pressure is more prominent and where prevalence of *P. malariae* is higher compared to Southeast Asia. The nonsynonymous mutations identified in PmDHPS and PmDHFR require further *in vivo* and *in vitro* studies to elucidate their significance in SP resistance in *P. malariae*.
